# The Complete Genome and Physiological Analysis of the Microbialite-Dwelling *Agrococcus pavilionensis* sp. nov; Reveals Genetic Promiscuity and Predicted Adaptations to Environmental Stress

**DOI:** 10.3389/fmicb.2018.02180

**Published:** 2018-10-15

**Authors:** Richard Allen White, Greg Gavelis, Sarah A. Soles, Emma Gosselin, Greg F. Slater, Darlene S. S. Lim, Brian Leander, Curtis A. Suttle

**Affiliations:** ^1^Department of Microbiology and Immunology, University of British Columbia, Vancouver, BC, Canada; ^2^Department of Zoology, University of British Columbia, Vancouver, BC, Canada; ^3^School of Geography and Earth Sciences, McMaster University, Hamilton, ON, Canada; ^4^Department of Earth, Ocean and Atmospheric Sciences, The University of British Columbia, Vancouver, BC, Canada; ^5^Bay Area Environmental Research Institute, Petaluma, CA, United States; ^6^NASA Ames Research Center, Moffett Field, CA, United States; ^7^Canadian Institute for Advanced Research, Toronto, ON, Canada

**Keywords:** actinobacteria, microbialite, cosmopolitan, oligotrophic, metabolic potential

## Abstract

Members of the bacterial genus *Agrococcus* are globally distributed and found across environments so highly diverse that they include forests, deserts, and coal mines, as well as in potatoes and cheese. Despite how widely *Agrococcus* occurs, the extent of its physiology, genomes, and potential roles in the environment are poorly understood. Here we use whole-genome analysis, chemotaxonomic markers, morphology, and 16S rRNA gene phylogeny to describe a new isolate of the genus *Agrococcus* from freshwater microbialites in Pavilion Lake, British Columbia, Canada. We characterize this isolate as a new species *Agrococcus pavilionensis* strain RW1 and provide the first complete genome from a member of the genus *Agrococcus.* The *A. pavilionensis* genome consists of one chromosome (2,627,177 bp) as well as two plasmids (HC-CG1 1,427 bp, and LC-RRW783 31,795 bp). The genome reveals considerable genetic promiscuity via mobile elements, including a prophage and plasmids involved in integration, transposition, and heavy-metal stress. *A. pavilionensis* strain RW1 differs from other members of the *Agrococcus* genus by having a novel phospholipid fatty acid iso-C15:1Δ^4^, β-galactosidase activity and amygdalin utilization. Carotenoid biosynthesis is predicted by genomic metabolic reconstruction, which explains the characteristic yellow pigmentation of *A. pavilionensis*. Metabolic reconstructions of strain RW1 genome predicts a pathway for releasing ammonia via ammonification amino acids, which could increase the saturation index leading to carbonate precipitation. Our genomic analyses suggest signatures of environmental adaption to the relatively cold and oligotrophic conditions of Pavilion Lake microbialites. *A. pavilionensis* strain RW1 in modern microbialites has an ecological significance in Pavilion Lake microbialites, which include potential roles in heavy-metal cycling and carbonate precipitation (e.g., ammonification of amino acids and filamentation which many trap carbonate minerals).

## Introduction

Microbialites represent the oldest evidence of life on the planet with fossils dating back to around 3.7 billion years ago (Nutman et al., 2016). These structures consist of a specialized microbial mat that lithifies carbonates into two main structural types, (1) thrombolites composed of non-laminated clots, or (2) stromatolites defined by laminated layers (Burne and Moore, 1987; Perry et al., 2007). Microbialites are still present today and represent natural laboratories of early microbial ecosystems, which allow for testing hypotheses around the basic principles of microbial ecology including questions regarding community composition (Wong et al., 2015, 2017), community assembly (Havemann and Foster, 2008), functional traits, and diversity (Breitbart et al., 2009; Saghaï et al., 2015; White et al., 2015, 2016b; Ruvindy et al., 2016; Louyakis et al., 2018) and the discovery of novel taxa (Burns et al., 2012).

While heterotrophs and photoautotrophs—mainly cyanobacteria—have been described and isolated from a range of microbial mats, including microbialites, little work has been done on pigmented heterotrophic bacteria within microbialites. It has been suggested that a variety of pigments could come from non-phototrophic bacteria (Nübel et al., 1999; Lionard et al., 2012). Actinobacteria have also been identified in the pigmented layers in microbial mats (Bottos et al., 2008; Lionard et al., 2012), and it is thought that carotenoids are responsible for their characteristic coloration (Nübel et al., 1999; Mueller et al., 2005; Klassen, 2010). Given that these groups seemingly lack metabolism for oxygenic or anoxygenic photosynthesis, fundamental questions include (1) what is the function of pigmentation in these heterotrophic bacteria? And (2) what are the potential roles of heterotrophic bacteria in cold microbialites or microbial mats? We enriched and isolated >100 pigmented bacteria from microbialites in Pavilion Lake, in southeastern British Columbia, Canada (50.8°N, 121.7°W). Based on their growth in the dark, they were either mixotrophs or heterotrophs. Among our enrichments the one isolate described here; which belongs to the *Agrococcus* genus and is a Gram-positive member of the phylum *Actinobacteria*.

Pavilion Lake is a cold, oligotrophic ecosystem (mean total phosphorus, 3.3 μg L^-1^), with dimictic, circumneutral waters (median pH 8.3; mean calcium carbonate, 182 mg L^-1^) (White et al., 2016b). Characterization of the limnology of Pavilion Lake is described in detail Lim et al. (2009). Pavilion Lake microbialites are calcium carbonate-based thrombolites with thin (∼5 mm) microbial mats dominated by cyanobacteria that change morphology as a function of lake depth (White et al., 2016b). Characterization of the limnology of Pavilion Lake is described in detail Lim et al. (2009). Our *Agrococcus* strain was isolated and enriched from a Pavilion microbialite (i.e., a thrombolite) at 20 m depth, where the water temperature remains around 4 to 10°C throughout the year (Lim et al., 2009). Bacteria at this depth should be adapted to cold temperature, low phosphorus, and alkaline conditions.

The genus *Agrococcus* was described based on two strains of *Agrococcus jenensis* isolated from soil and the surface of sandstone (Groth et al., 1996). The genus *Agrococcus* is classified within the family *Microbacteriaceae*, within the phylum *Actinobacteria.* All *Agrococcus* members have diaminobutyric acid within their cell walls (Groth et al., 1996). Diaminobutyric acid may impart the distinctive lemon-yellow color, although its role in pigmentation is unknown (Groth et al., 1996). *Agrococcus* spp. have been isolated from a wide range of environments, including air (Zlamala et al., 2002), a coal mine (Dhanjal et al., 2011), cheese (Bora et al., 2007), cold-desert soil (Mayilraj et al., 2006), forest soil (Zhang et al., 2010), a medieval wall painting (Wieser et al., 1999), dried seaweed (Lee, 2008), and the phyllosphere of potato plants (Behrendt et al., 2008). There are eight described species of *Agrococcus*, yet little is known about the genome, metabolism, evolution, or physiology of this genus.

To explore the potential role of this genus in microbialite communities, we herein characterize the new species *Agrococcus pavilionensis* strain RW1, using both classical bacteriological examination (e.g., chemotaxonomic investigation of its metabolism) along with modern genome-centric approaches. We provide the first complete reference genome (i.e., closed gapless chromosome with two plasmids) from the *Agrococcus* genus. Our genomic analyses suggest signatures of environmental adaption to the relatively cold and oligotrophic conditions of Pavilion Lake microbialites. Promiscuous mobile elements were found in two plasmids involved in heavy-metal resistance and DNA transposition. The genomes of *A. pavilionensis* RW1 and *A. lahaulensis* K22-21 both encode a carotenogenic gene cluster that could be responsible for producing the characteristic lemon-yellow pigmentation found in isolates of *Agrococcus spp.* We also discuss further the potential roles of *A. pavilionensis* RW1 in microbialite formation.

## Materials and Methods

### Isolation, Growth Conditions, Microscopy, Phage Induction, Biochemical and Antibiotic Susceptibility Tests

**FIGURE 1:**
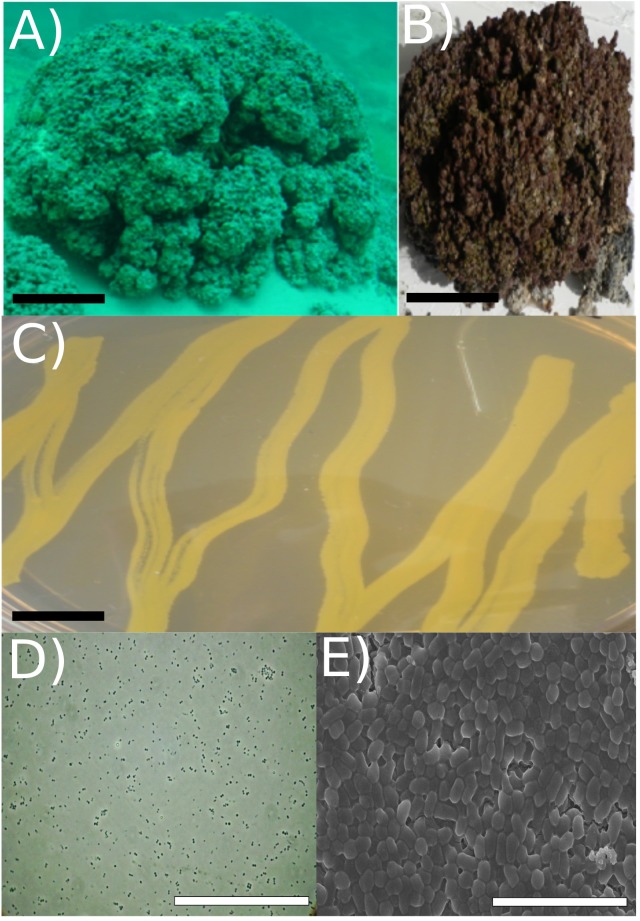
Strain RW1 isolation environment, culture plate illustration, and microscopy. **(A)** Picture of Pavilion Lake microbialite (i.e., thrombolite) taken underwater (20 m depth sample, scale bar: ∼1 m). **(B)** Pavilion Lake microbialite (i.e., thrombolite) taken at sample station (20 m depth sample, scale bar: ∼15 cm). **(C)** Culture plate (∼24 h growth, the black scale bar ∼10 mm). **(D)** Light microscopy (∼72 h growth, scale bar: ∼80 μm); **(E)** SEM (∼72 h growth, scale bar: ∼5 μm). All microscopy (i.e., SEM/light) and culture plate photo (C-E) were completed at strain RW1 standard growth parameters on solidified LB-agar at 30°C, pH 7 and concentration 1% (w/v) NaCl.

*Agrococcus pavilionensis* strain RW1 was isolated by plating 0.5 g of a thrombolytic microbialite collected from a depth of 20 m in Pavilion Lake, British Columbia (50.86677 °N, 121.74191 °W). Plating occurred in lysogeny broth (LB) [1% (w/v) tryptone, 0.5% (w/v) yeast extract, 1% (w/v) NaCl, pH 7], and incubated at 10°C for 1 week as an enrichment culture (**Figure 1**). The enrichment culture was selected for pigmented single colonies then were streaked for isolation on LB agar plates [solidified with 1.5% (w/v) agar] at 30°C for 3 days. Solidified LB agar was also used for assessing growth at various temperatures (4, 11, 16, 20, 25, 30, 35, 42, and 50°C), at pH 7 and 1% (w/v) NaCl. To assess the effect of pH (5, 6, 6.5, 7, 7.5, 8, 8.5, 9, 10, 10.5, and 11) on growth, standard solidified LB was used at 30°C and at a concentration of 1% (w/v) NaCl. To test the effect of salinity on growth, different amounts of NaCl were added to achieve final salinities of 0–6, 9, 12, 13, and 16% on solidified LB (pH 7). The cultures were grown at 30°C and maintained in LB medium or on agar at 1% NaCl, pH 7 and at 30°C. M-agar medium [0.5% (w/v) tryptone, 0.25% (w/v) yeast extract, 1% (w/v) NaCl, 1.5% (w/v) agar, pH 7]. A diluted version of LB agar medium, was used to determine filamentous growth under carbon-limitation. Strain characteristics, including colony morphology and cell morphologies, were determined by standard methods (Murry et al., 1994). Oxidase tests, biochemical enzyme assays, and carbohydrate utilization tests were completed using API20E (BioMérieux) test strips on cultures re-suspended in sterile water. Antibiotic susceptibility of strain RW1 was determined by the Kirby-Bauer method using antibiotic disks on solidified LB (Collee et al., 1996).

Prophage induction assays were done either by adding 0.2 μg ml^-1^ of mitomycin C or by heating cultures to 45°C for 5 min, incubating at 30°C for 3–10 h, and monitoring changes in turbidity until a decrease of OD_600_ to 0.1 or less (starting OD_600_ = 0.6). At several time points (3, 5, and 10 h), cells were pelleted at 3,250 × g and the supernatant filter-sterilized twice through a 0.22 μm pore-size Millex Durapore PVDF membrane (Millipore) filters before plating using a double agar overlay plaque assay (Kropinski et al., 2009).

Light and scanning electron microscopy (SEM) was completed on exponentially growing cells in LB medium. Cells were harvested after 48 h and viewed under oil immersion at 1,500 times magnification for light microscopy. For SEM, cultures in liquid LB were filtered at 72 h onto a 0.2 μm pore-size Supor polycarbonate membrane (Pall, Port Washington, NY, United States). Cells were fixed in 2.5% glutaraldehyde in phosphate-buffered saline (PBS) [137 mM NaCl, 2.7 mM KCL, 10 mM Na_2_HPO_4_2H_2_O, 2 mM KH_2_PO_4_, pH 7.4] for 30 min on ice. Cells were washed with PBS and post-fixed in 1% OsO_4_ for 1 h. Stained cells were passed through a graded ethanol series (25, 50, 70, 95, and 100%) at 10-min intervals, and critical-point dried in 100% EtOH. A sputter coater applied 5 nm of gold/palladium alloy onto the cells before imaging by SEM using a Hitachi S4700 microscope.

### Chemotaxonomic Analysis of Phospholipids

Phospholipid fatty acids (PLFAs) were extracted from cultures grown in 25 ml LB medium for 4 days at 22°C using triplicate biological replicates. Cultures were transferred into pre-combusted vials for an overnight solvent extraction in a 1:2:0.8 ratio of dichloromethane (DMC): methanol (MeOH): PBS [137 mM NaCl, 2.7 mM KCL, 10 mM Na_2_HPO_4_ 2H_2_O, 2 mM KH_2_PO_4_, pH 7.4] solution (Bligh and Dyer, 1959). The extract was filtered through a separatory funnel where DCM and water were added to achieve a mixture of MeOH-:DCM:water of 1:1:0.9 (Bligh and Dyer, 1959). The lower organic phase was removed and purified into polar, neutral, and non-polar fractions using liquid chromatography through silica gel. Phospholipids present in the polar fraction were subjected to mild alkaline methanolysis to produce fatty acid methyl esters (FAMEs) (Guckert et al., 1985). FAMEs were separated, identified, and quantified using gas chromatography mass spectrometry (GC/MS) (Agilent Technologies Inc., Santa Clara, CA, United States) with a DB-5MS capillary column (30 m × 0.32 mm I.D. ×0.25 μm film thickness) at a temperature regime of 50°C (1 min), 20°C min^-1^ to 130°C, 4°C min^-1^ to 160°C, and 8°C min^-1^ to 300°C (5 min). PLFAs were identified by retention time and mass spectra relative to those of reference standards (Bacterial Acid Methyl Ester Mix, Matreya Inc., Pleasant Gap, PA, United States; and Supelco 37 Component FAME Mix, Sigma-Aldrich Co., Bellefonte, PA, United States). A modified picolinyl ester derivatization was used to determine the branching point in unknown compounds (Dowd, 1998; Destaillats and Angers, 2002). Dimethyl disulfide adduct derivatives were prepared to determine the double-bond position in unsaturated fatty acids (Nichols et al., 1986).

### DNA Extraction, PCR, and Illumina Library Construction

DNA was extracted from early log-phase colonies of *Agrococcus pavilionensis* strain RW1 grown on LB agar plates using a QIAamp DNA Mini Kit, followed by MinElute PCR purification cleanup columns (Qiagen Germantown, MD, United States). We amplified 16S rRNA by using the universal primers 27f and 1492r (Lane, 1991), and a second PCR using primers 341f and 907r to obtain sequence overlap between the 27f and 1492r to complete the full-length 16S rRNA gene (Muyzer et al., 1993; Muyzer and Smalla, 1998). PCR products were sequenced using standard Sanger method on an ABI3730 (Applied Biosystems, Foster City, CA, United States). The Illumina MiSeq library was constructed using the NxSeq Library Prep Kit (Lucigen, Middleton, WI, United States) without the final 14-cycle PCR enrichment to avoid PCR bias. Quality control of the resulting library was completed using Agilent high-sensitivity DNA chips and digital droplet PCR (Hindson et al., 2011; White and Suttle, 2013; White et al., 2013a,b).

### Phylogenetic Analysis

Sanger sequences obtained from the 27f-1492r and 341f-907r PCR products were merged into a full-length 16S rRNA gene sequence using Consed (Gordon et al., 1998) with manual editing. BLAST analysis of both the full-length PCR product and the whole-genome assembled 16S rRNA gene suggested that our culture was a member of the *Agrococcus* genus. The phylogenetic position of *A. pavilionensis* strain RW1 was assessed using the error-corrected whole-genome assembled 16S rRNA gene (∼99% similar to PCR amplified) rather than the PCR amplified sequence.

Multiple locus sequencing typing (MLST) marker analysis was completed by extracting protein sequences from rpoB (β subunit of bacterial RNA polymerase, ∼1156 amino acids), RecA (recombination protein A, ∼352 amino acids), gyrB (DNA gyrase subunit B, ∼679 amino acids), and ppK (Polyphosphate kinase, ∼752 amino acids, from both draft and completed genomes by BLASTP analysis or from prior MLST analysis (*A. jenensis* strain DSM9580 only, Stackebrandt et al., 2007) then concatenated into a ∼2939 amino acid sequence. All phylogenetic analyses were aligned using muscle-default parameters (-400 gap open with zero gaps extended) then clustered using UPGMB. Trees were then constructed using maximum likelihood with bootstrapping (1000 replicates) and the Jukes-Cantor substitution model for 16S rRNA gene full-length sequences (as default parameters), and Jones-Taylor-Thronton model (as default parameters) for ∼2939 amino acid MLST concatenated sequences in MEGA (Edgar, 2004; version 5.10, Tamura et al., 2011).

### Whole-Genome Assembly and Genome Finishing

Read-error correction and Celera assembly (including plasmid pHC-CG425) and read partitioning were done as described (White et al., 2013b). Ray assembly of the bacterial genome using the error-corrected reads and phiX removal were done as described (White et al., 2013b,c).

A method to align two or more genomes, progressiveMauve, was used to find the best representative assembly and contig order, and to complete the genome (Darling et al., 2010). Contigs from Celera and Ray assemblies were pooled, then the remaining gaps were closed by recursive alignments in Mauve. The draft *A. lahaulensis* genome from NCBI (version ASM42510v1) was used for genome ordering. The ordered and aligned overlapping contigs were merged using the EMBOSS union script, yielding three circular contigs (Rice et al., 2000).

To confirm the three circular contigs as separate circular genomes, read-mapping was used. Error-corrected, phiX-removed reads were mapped back to the genome and plasmids using Bowtie2 (version 2.3.4) with the very sensitive local option (Langmead and Salzberg, 2012). The Bowtie2 read-mapping output file (Sam file) was visually inspected by the Tablet program (Milne et al., 2013).

Annotation was completed on RAST using SEED (Aziz et al., 2008); RAST server parameters used SEED subsystems with FIGfam under the Glimmer 3 option (Meyer et al., 2009). In addition to RAST, metabolic pathways were predicted using MetaPathways, a modular pipeline for gene prediction and annotation that uses pathway tools and the MetaCyc database to construct environmental pathway/genome databases (ePGBDs) (Paley and Karp, 2006; Konwar et al., 2013; Caspi et al., 2014).

Annotations were further analyzed for comparison to the *A. lahaulensis* strain K22-21 and analyzed for genome synteny, average amino acid identity, and phage lifestyle prediction. The genome circular plot was constructed using CGViewer (Grant and Stothard, 2008). *Agrococcus lahaulensis* strain K22-21, Celera (k0-k1250), and Ray (k0-k1250v2) assemblies were mapped to the completed genome of the *A. pavilionensis* strain RW1 using tBLASTx at an Expect (E) value of 1e^-3^ with 50% identity and 25 bp overlap. Synteny plots were completed in the RAST server module using a BLAST-based dot plot format (Aziz et al., 2008). Average amino acid identity (AAIr) analysis and functional gene similarities were calculated on the RAST server module, then parsed by a web-based tool (Aziz et al., 2008; Krebs et al., 2013). RAST-server annotation predicted a prophage element in the genome, which was analyzed for lifestyle preference (lytic or lysogenic) using the phage classification toolset (PHACTS) (McNair et al., 2012).

FR-hit program was used for metagenomic recruitment for the *Agrococcus* genomes using default parameters with a minimum identity >70% and an Expect (E) value >1e^-5^ (Niu et al., 2011). The recruitments were then visualized with the R library ggplot2 (Wickham, 2009).

### Data Availability

*Agrococcus pavilionensis* strain RW1 is listed at NCBI under BioProject accession PRJNA201450. The Celera assembly (k0-k1250) of *Agrococcus pavilionensis* RW1 is under RefSeq NZ_ASHR00000000.1 and assembly GCF_000400485.1 at NCBI. The *Agrococcus lahaulensis* strain K22-21 is listed at NCBI under BioProject accession PRJNA188801.

## Results and Discussion

### Morphology and Growth Characteristics

The cells were coccoid during log phase (∼48 h) and were irregular rod-like or coccoid in stationary phase (∼72 h) (**Figure 1**). The cell size of *A. pavilionensis* strain RW1 was 0.5 to 0.7 μm in diameter, which is similar to other described members of the genus (Zhang et al., 2010; **Table 1**). On solidified LB, colonies were bright yellow, smooth and circular, and were typically 0.5 to 2 mm in diameter after ∼72 h of growth at 30°C.

**Table 1:** Comparison of physiological properties of selected strains of *Agrococcus* spp. – No growth (-), Growth (+), Data not available (NA), Temperature (temp).

	Strain RW1	*A. lahaulensis*: *K22-21*	*A. baldri-V-108*	*A. citreus^1^* *D-l/la*	*A. jenensis^1^* *2002-39/1*	*A. terreus^1^* *DNG5*
Habitat	Microbialite	Desert soil	Air	Painting	Sandstone	Forest soil
**Cell size (μm)**						
Length	0.5–0.7	1.0–1.5	1.1–1.7	1.1–1.7	0.7–1.7	0.8–1.0
Width	0.3–0.5	0.6–1.0	0.7–1.0	0.7–1.0	0.7–1.0	0.4–0.5
**Growth range(°C)**						
11	+	NA	NA	NA	NA	NA
30	+	+	+	+	+	+
37	+	+	W	+	V	+
42	+	-	-	NA	-	NA
**pH growth**						
6	+	+	NA	NA	NA	+
7	+	+	+	+	+	+
10	+	+	NA	NA	NA	-
**NaCI tolerance**						
0%	+	NA	NA	NA	NA	+
6%	+	+	+	+	NA	-
7%	-	+	NA	+	NA	-

*All growth measurements for *A. pavilionensis* strain RW1 were taken after 3 days. ^*1*^Groth et al. (1996); Wieser et al. (1999); Zlamala et al. (2002); Zhang et al. (2010). Strain RW1 was measured directly on LB agar plates if colonies were present >100 growth (+) was used and if no colonies were present (-) was used. Weak growth (W) and variable growth (V) were described for two observations in the various references strains in there various manuscripts but were listed as no growth (-) in our table*.

The morphology of *A. pavilionensis* strain RW1 shared features with other members of the genus but had a novel phenotype of filament-like growth. This growth form emerged in low-carbon conditions (i.e., diluted LB or M-agar), and had pale-yellow to white colonies with irregularly branching filaments. These could potentially act as nucleation points for carbonate precipitation within the Pavilion Lake microbialite mat. However, further study of carbonate precipitation and nucleation on *Agrococcus pavilionensis* strain RW1 is ongoing. While filamentous growth morphology is common among actinobacteria (e.g., isolates of *Streptomyces* spp.), this phenotype has not been reported for other members of the *Microbacteriaceae* (Doroghazi and Metcalf, 2013). Further experimentation is needed to confirm whether this is a unique adaptation of *A. pavilionensis* strain RW1 to microbialites or whether this phenotype is more widely spread across the genus of *Agrococcus* under low-carbon conditions.

*Agrococcus pavilionensis* strain RW1 grows on under many conditions. Growth occurred from pH 6 to 10, at 0 to 6% added NaCl, and over a temperature range of 11 to 42°C on LB agar (**Table 1**). A close relative, *A. lahaulensis* strain K22-21, has a narrower temperature range of growth (between 30 and 37°C) but can grow at salt concentrations as high as 7% (Mayilraj et al., 2006; **Table 1**). Contrary to expectations for a cold-water isolate, *Agrococcus pavilionensis* strain RW1 had the highest reported growth temperature for the genus (Zhang et al., 2010), at 42°C, and had no observed growth at or below 4°C (**Table 1**), while it exhibited slow growth at 10°C. This suggests that growth of *A. pavilionensis* RW1 within microbialites may be seasonal. Water temperatures in Pavilion Lake at 20 m range from 4 to 10°C throughout the year (Lim et al., 2009).

### PLFA Characterization and Comparative Analysis

Phospholipid fatty acid is commonly used to distinguish bacterial isolates in classical bacterial strain naming. The PLFA composition of *A*. *pavilionensis* RW1 was distinct from other strains of *Agrococcus* spp., including its close relative *A. lahaulensis. Agrococcus pavilionensis* RW1 had half the amount of iC16:0 but three times as much C16:0 compared to *A. lahaulensis* (Mayilraj et al., 2006; **Table 2**). The branched unsaturated PLFA iC15:1Δ^4^ was 3.5% of the total PLFAs found in *A*. *pavilionensis* RW1. It was only found in trace amounts (<1%) in *A. versicolor* strain K 114/01^T^ (Behrendt et al., 2008; **Table 2**). Branched monoenoic PLFAs such as iC15:1Δ^4^ are typically used as biomarkers for anaerobic sulfate-reducing bacteria. Yet *A*. *pavilionensis* RW1 grows aerobically and does not reduce sulfate (Kohring et al., 1994). Branched PLFAs found in *A*. *pavilionensis* RW1 are known biomarkers for Gram-positive bacteria (Kaur et al., 2005). Although the PLFA profiles between *A*. *pavilionensis* RW1 and *A. lahaulensis* K22-21 are quite similar, the differences support the phylogenetic inference that the two isolates are from different taxonomic groups.

**Table 2:** Selected cellular phospholipid fatty acids of selected strains of *Agrococcus* spp.

	Strain RW1	*A. lahaulensis^1^* *K22-21*	*A. baldri^1^* *V-108*	*A. citreus^1^* *D-1/1a*	*A. jenensis^1^* *2002-39/1*
C14:0	ND	ND	ND	tr	tr
iC14:0	ND	ND	ND	tr	tr
C15:0	ND	ND	ND	tr	ND
iC15:Δ4	3.5 ± 0.1	ND	ND	ND	ND
iC15:l	ND	ND	ND	ND	1.9
aiC15:l	ND	tr	ND	ND	0
iC15:0	8.8 ± 0.2	9.9	5.7	10	12.2
aiC15:0	46.6 ± 2.4	48.4	44.9	53.1	57.8
C16:0	5.5 ± 0.7	1.8	3	1.7	2
iC16:0	2.8 ± 0.6	5.8	7.5	12	12.6
iC17:0	3.1 ± 0.5	4.8	1.5	1.7	1.9
aiC17:0	29.7 ± 1.0	27.6	24.3	13.2	9.3
aiC17:l	ND	ND	ND	ND	ND
C18:0	ND	ND	ND	tr	tr

*Values are percentages of total phospholipid fatty acids. Not detected (ND), Trace (<1%, tr). Strain RW1: *A. pavilionensis*^*1*^Mayilraj et al. (2006)*.

Unsaturated branched PLFAs found in *A*. *pavilionensis* RW1 may be a survival adaptation to cold temperatures present in Pavilion Lake since unsaturated fatty acids are used to compensate for a decrease in membrane fluidity found at cold temperatures (Los and Murata, 2004). *A. lahaulensis* was isolated from cold soil in Lahaul-Spiti Valley in the Indian Himalayas, which also contains unsaturated, branched PLFAs (Mayilraj et al., 2006). These features of unsaturated, branched PLFAs may therefore facilitate the adaption to colder temperatures of both *A*. *pavilionensis* and *A. lahaulensis.*

### Evolutionary Placement of *Agrococcus pavilionensis* Strain RW1

**FIGURE 2:**
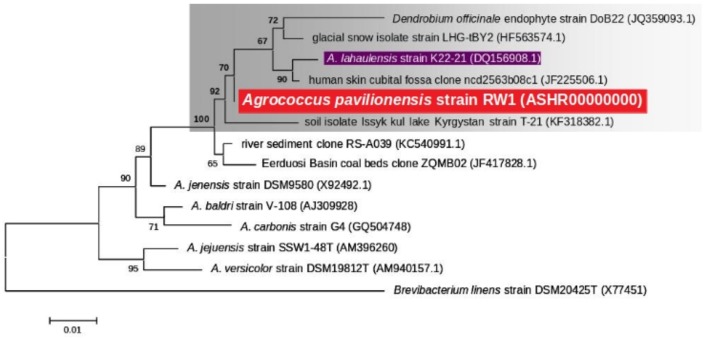
Maximum likelihood tree based on 16S rRNA gene sequences (∼1409 bp) showing the phylogenetic relationship among the different isolates of the genus *Agrococcus.* Bootstrap values greater than 50% are given at nodes. Bar represents a nucleotide substitution rate per 100 nucleotides.

**FIGURE 3:**
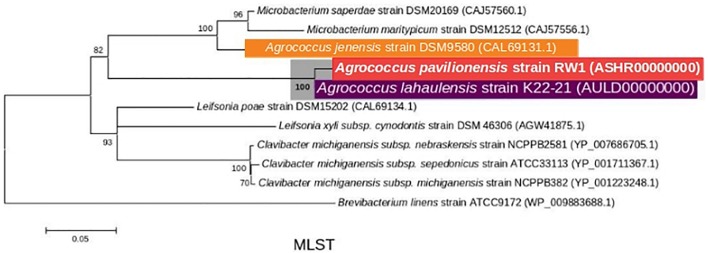
Maximum-Likelihood tree based on MLST analysis (∼2939 amino acid) showing the phylogenetic relationship among the different isolates of the genus *Agrococcus* and deeper relatives of the *Microbacteriaceae.* Bootstrap values greater than 50% are given at nodes. Bar represents a nucleotide substitution rate per 100 nucleotides.

Phylogenetic analysis of the 16S rRNA gene indicates that *A. pavilionensis* RW1 was most closely related to a clade containing *A. lahaulensis* K22-21, and an isolate from human-skin (**Figure 2**). However, a full-length 16S rRNA sequence alone was unable to resolve whether *A. pavilionensis* RW1 and *A. lahaulensis* are different species. MLST analysis suggests that *A*. *pavilionensis* strain RW1 and *A. lahaulensis* are in the same clade, but was unable to resolve whether they are separate species (**Figure 3**). MLST needs a minimum of seven loci to assign a species-level classification of closely related bacterial species. Only four loci are available for the genus *Agrococcus* (Maiden et al., 2013), so speciation could not be assigned by MLST alone.

**FIGURE 4:**
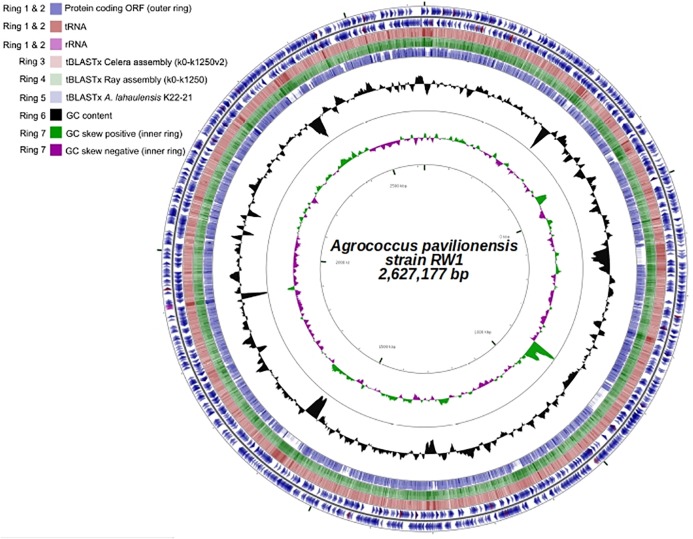
Genome plot (∼2.6 Mb) of *A. pavilionensis* strain RW1. Genome key (left corner): starts with the innermost ring, which is a genome ruler followed by GC skew (purple/green) and ends with two outer rings, which contain protein coding ORFs, tRNAs, and rRNAs.

**FIGURE 5:**
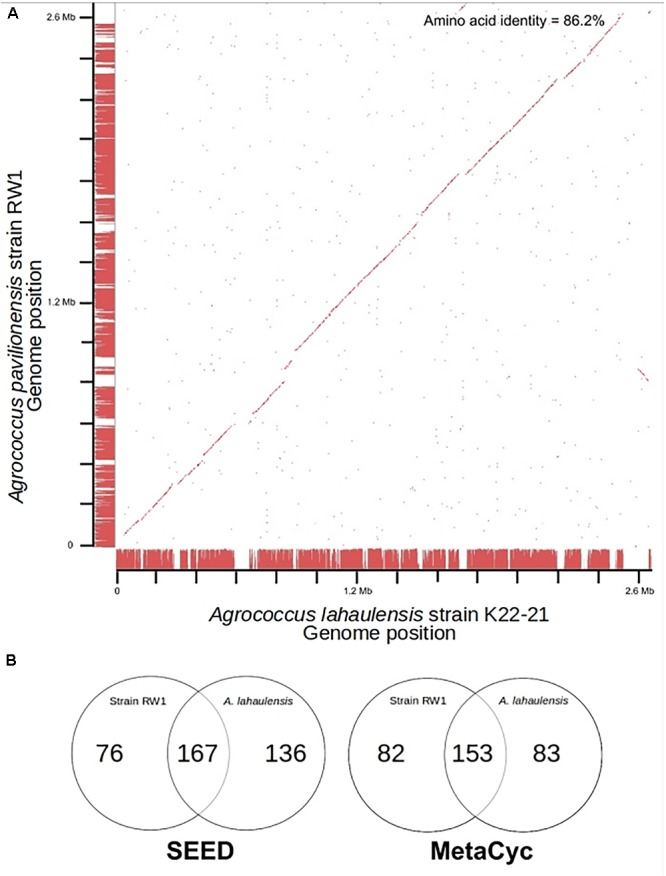
Genome synteny and Venn diagrams of *A. pavilionensis* RW1 vs. *A. lahaulensis* K22-21. **(A)** RAST bidirectional BLAST genome synteny dot plot with Progressive Mauve alignments as axes. Red dots are positive BLAST hits based on the RAST genome comparison module. Red lines at the axes are regions of synteny based on Progressive Mauve. Amino acid identity was calculated by RAST functional module with a web-based tool (Krebs et al., 2013). **(B)** Venn Diagrams based on RAST SEED/FIGfams and Metapathways MetaCyc annotations.

Fortunately, a draft genome is available for *A. lahaulensis*, making it possible to infer their relationship based on an analysis of synteny between the two genomes. We mapped the assemblies of *A. lahaulensis* K22-21 and *A*. *pavilionensis* RW1 (both Ray and Celera) against the final circular chromosome of *A*. *pavilionensis* RW1 using tBLASTx. Only the *A. lahaulensis* assembly showed gaps (**Figure 4** and **Supplementary Table S1**). Synteny plots revealed 12 large gaps between the genomes of *A*. *pavilionensis* RW1 and *A. lahaulensis*, K22-21, along with 1752 non-conserved intergenic regions in *A. lahaulensis* (**Figure 5**). A comparison of functional gene annotations for *A*. *pavilionensis* RW1 and *A. lahaulensis* K22-21, using both SEED (RAST-based) and MetaCyc (MetaPathways-based), revealed >200 conserved genes, demonstrating that only a small core genome is conserved between the isolates (**Figure 5**). Thus, while they are closely related, it seems that their subclade within the genus shows high genomic plasticity.

Average amino acid identity between the two genomes supports classifying *A*. *pavilionensis* RW1 and *A. lahaulensis* K22-21 as different species. This is a robust measure for bacterial species classification based on whole-genome sequences and comparable to DNA-DNA hybridization (Konstantinidis and Tiedje, 2005). The standard cutoff to distinguish isolates as different bacterial species is <70% similarity by DNA-DNA hybridization. This corresponds to <95% average amino acid identity (Konstantinidis and Tiedje, 2005). The average amino acid identity for *A*. *pavilionensis* RW1 and *A. lahaulensis* K22-21 was only 86.2%, based on bidirectional whole-genome best-hit protein analysis using RAST annotation. That supports the classification of the isolates as different species (Konstantinidis and Tiedje, 2005; Krebs et al., 2013; **Figure 5**).

### Biochemical Properties and Antibiotic Susceptibility

*Agrococcus pavilionensis* RW1 shared many biochemical properties with other members of the genus. That included being Gram-positive, but negative for oxidase, arginine dihydrolase, lysine decarboxylase, ornithine decarboxylase, urease, use of citrate and inositol/myo-inositol, production of hydrogen sulfide, and indole and acetoin and positive for catalase (Behrendt et al., 2008). In contrast, *A. pavilionensis* RW1 was positive for β-galactosidase activity, whereas other isolates have only weak or no activity (Behrendt et al., 2008; **Table 3**), and *A. lahaulensis* has no predicted β-galactosidase activity or corresponding genes (Mayilraj et al., 2006; **Table 3**). Given the diversity of galactosidases in other microbial mat-dwelling heterotrophs, it is possible that β-galactosidase allows *A. pavilionensis* RW1 to digest exopolysaccharides or other carbohydrates within the mat, though this remains to be tested (Leyn et al., 2017).

**Table 3:** Biochemical properties of *Agrococcus* selected strains.

	Strain RW1	*A. lahaulensis*: *K22-21*	*A. baldri*: *V-108*	*A. citreus^1^* *D-l/la*	*A. jenensis^1^* *2002-39/1*	*A. versicolor^1^* *K114/01(T)*
**Hydrolysis of:**						
Gelatinase	-	+	-	-	-	ND
**Activity of:**						
β-galactosidase	+	-	-	-	-	W
**Assimilation of:**						
D-glucose	+	-	+	-	+	+
D-mannitol	+	+	+	+	+	W
D-sorbitol	+	-	+	-	-	-
L-Rhamnose	+	+	-	-	-	-
D-sucrose	+	-	-	-	-	+
Amygdalin	+	-	-	-	-	-
L-arabinose	-	+	+	+	+	+

*Not detected (ND), Weakly positive (W), Strain RW1: *Agrococcus pavilionensis*. ^*1*^Behrendt et al., 2008*.

Tests for antibiotic sensitivity in *A. pavilionensis* RW1 show a pattern that is similar to other isolates of *Agrococcus* spp., including being sensitive to penicillin, tetracycline, streptomycin, and rifampin (Wieser et al., 1999; **Table 4**). It is also sensitive to tobramycin, vancomycin, and clindamycin, but resistant to cefixime, sulfisoxazole, oxacillin, trimethoprim and a mixture of sulfamethoxazole/trimethoprim, antibiotics for which patterns of resistance in other strains are less clear, or for which comparable data are not available (**Table 4**). *Agrococcus citreus* and *A. jenensis* strain DSM9580^T^ and DSM9996 are sensitive to oxacillin at 5 μg and weakly sensitive to polymyxin (Wieser et al., 1999); whereas *A. pavilionensis* RW1 was resistant to oxacillin at 1 μg and sensitive to polymyxin (**Table 4**). The resistance of *A. pavilionensis* RW1 to 1 μg of oxacillin, while other *Agrococcus* spp. are sensitive to doses of 5 μg, suggests that *A. pavilionensis* RW1 could be sensitive to higher oxacillin concentrations (Wieser et al., 1999). β-lactamase is commonly involved in oxacillin resistance, but evidence for its occurrence was not found in the genomes of either *A. pavilionensis* RW1 or *A. lahaulensis* K22-21 (Hou et al., 2007). Although there were no putative antibiotic resistance genes predicted within the genome of *A. pavilionensis* RW1, pathways were predicted for aromatic compound degradation, including salicylate and gentisate catabolism, which may be involved in resistance. It is conceivable that this antibiotic resistance may be necessitated by exposure to toxic organic molecules produced by cyanobacterial mats, and that it may act as a survival mechanism (Neilan et al., 2013). To better understand antibiotic resistance in *Agrococcus* spp., more isolates should be tested in future studies.

**Table 4:** Antibiotic susceptibility of selected strains of *Agrococcus* spp.

Antibiotic	Disk content	Strain RW1	*A. citreus^1^* *Dl/1aT*	*A. jenensis^1^* *DSM9580T*
Sulfamethoxazole + Trimethoprim	23.75 + 1.25 μg	+	ND	ND
Penicillin	10 IU	-	-	-
Clindamycin	2 μg	-	ND	ND
Rifampin^μ^	5/30 μg	-	-	-
Polymyxin	300 IU	-	W	W
Cefixime	5 μg	+	ND	ND
Sulfisoxazole	300 μg	+	ND	ND
Oxacillin^μμ^	1/5 μg	+	-	-
Tetracycline	30 μg	-	-	-
Trimethoprim	5 μg	+	ND	ND
Tobramycin	10 μg	-	ND	ND
Vancomycin	30 μg	-	ND	ND
Streptomycin	10 μg	-	-	-

*Resistant (+), Sensitive (-), Weakly Sensitive (W), No Data (ND). ^∗^*A. pavilionensis* Strain RW1 was sensitive to 5 μg, whereas the other strains were sensitive at 30 μg. ^∗∗^*A. pavilionensis* Strain RW1 was resistant to 1 μg, whereas the other strains were sensitive at 5 μg. ^*1*^Wieser et al. (1999)*.

### Mobile DNA and Viral Elements

**FIGURE 6:**
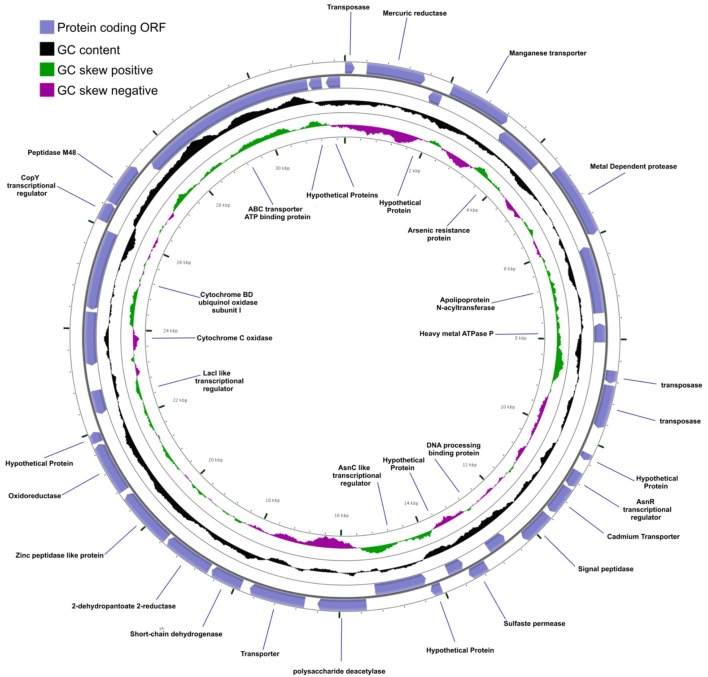
*Agrococcus pavilionensis* strain RW1 LC-RRW783 plasmid genomic plot. The blue lines are for the name of the predicted protein-coding open reading frame.

Mobile DNA elements, which are plasmid-encoded in *Agrococcus* sp. RW1, are predicted to function in integration, transposition, and heavy-metal resistance. Two plasmids (pHCCG425 and pLC-RRW783) discovered in *A. pavilionensis* RW1 are involved in integration, transposition, and heavy-metal resistance. The 1,427-bp plasmid pHC-CG425 has a GC content of 67.8% (4.7% less than the main chromosome) and two ORFs. One encodes a putative integrase, and the other encodes a hypothetical protein of unknown function. Meanwhile, pHC-CG425 plasmid shares strong similarities to gene clusters in other members of the phylum *Actinobacteria* including isolates of *Brevibacterium linens* and *Mycobacterium* spp. The second plasmid, pLC-RRW783, is 31,975 bp in length with a GC content of 70.6% (2% less than the main chromosome) and 36 ORFs (**Figure 6**), including putative coding sequences. Plasmid pLC-RRW783 contained ORFs annotated for mercuric reduction, arsenic resistance, various metal-dependent proteases, peptidases, ATPases, cadmium and unknown transporters, and an unclassified oxidoreductase. Seven ORFs in pLC-RRW783 have no predicted function and are annotated as hypothetical proteins.

Annotation of the genome of *A. pavilionensis* RW1 revealed a 34,174 bp prophage-like element that resembles *Siphoviridae* prophages found in actinobacteria. It has 43 predicted ORFs and a GC content of 70.1%, which is ∼2% less than the GC content of the genome (**Supplementary Figure S1**). The addition of heat and mitomycin C did not result in induction, suggesting the prophage is incapable of entering the lytic cycle, or that the treatments were not suitable inducing agents (Zheng et al., 2014). By comparison, no prophage or phage-like genes are predicted in the genome of *A. lahaulensis* K22-21. The prophage in *A*. *pavilionensis* RW1 has a predicted coding sequence for a phage tail-length tape measure protein and a phage-protease gene that are related to sequences found in other phages of *Siphoviridae.* Those include VWB, phi-c31, and phi-BT1, as well as the *Mycobacterium* phage Brujita, which were found infecting *Streptomyces* spp. (Gregory et al., 2003; Van Dessel et al., 2005).

### Nitrogen and Phosphorus Metabolism

*Agrococcus pavilionensis* RW1 encodes an incomplete ammonium utilization pathway that could also be involved in glutamine, glutamate, aspartate, and asparagine biosynthesis. It includes ORFs with similar coding sequences for glutamate-ammonia ligase adenyltransferase and for three NADPH glutamate synthase proteins. Glutamate-ammonia ligase adenyltransferase is conserved across related members in the phylum, including *Clavibacter michiganensis* and *Kocuria rhizophila.* It encodes about 1000 amino acids in length, whereas in *A. pavilionensis* RW1 it is truncated to 113 amino acids and is not predicted to be functional. The genomes of strain K22-21 and RW1 predicted ammonification of amino acids via aspartate, histidine, serine, glutamine, threonine ammonia lyases, and ammonium transporters. While both genomes encode a QacE-family quaternary ammonium compound efflux SMR transporter, strain RW1 encodes an extra copy. *Agrococcus pavilionensis* RW1 does release ammonium in late log phase or older cultures (via characteristic ammonium odor) Ammonium has been shown to increase carbonate biomineralization via ammonification through the deamination of amino acids (Rodriguez-Navarro et al., 2003). Further experimental confirmation is needed to ascertain if *Agrococcus pavilionensis* RW1 ammonification leads to carbonate biomineralization.

Genes related to those encoding the phosphate (Pho) regulon for high-affinity uptake of phosphate. Included were the phosphate permease protein (PstA), phosphate regulon sensor protein (PhoR), and the phosphate-regulon transcriptional regulatory protein (PhoB). The phosphate-regulon proteins (PhoR/PhoB) and PstA were not predicted within the *A. lahaulensis* K22-21 genome. Exopolyphosphatase, a purine metabolism enzyme, is predicted in both *A. lahaulensis* K22-21 and *A. pavilionensis* RW1. Polyphosphate glucokinase is only predicted in the *A. lahaulensis* K22-21 genome. Pavilion Lake is oligotrophic, with low concentrations of total phosphorus (3.3 μg L^-1^) (Lim et al., 2009). The phosphorus regulon (Pho) in the genome of *A. pavilionensis* RW1, which may be evidence of adaptation to low phosphate by encoding gene clusters linked to phosphorus regulation and acquisition. This machinery may represent an adaptation to the oligotrophic habitat where *A. pavilionensis* was found, since phosphate limitation has been found to actively induce the pho regulon in other bacteria (Suzuki et al., 2004). Indeed, genes associated with phosphorus adaptation and scavenging have also been found in metagenomic studies of other freshwater microbialites (Breitbart et al., 2009). Still, the response of the Pho regulon in *A. pavilionensis* RW1 under phosphorus limitation still has to be experimentally investigated.

### Life in a Cold and Oligotrophic Microbialite Mat

Pavilion Lake microbialites exist in water that ranges in temperature from 4 to 10°C (Lim et al., 2009). This frigid environment is reflected in the genome of *A. pavilionensis* RW1, which has signatures of cold adaptation. These signatures include a single copy of *cspA* that encodes cold-shock protein A (CspA), a protein that is induced at cold temperatures (<10°C) and which is essential for growth at <10°C. It functions as a sort of molecular chaperone that binds mRNA, preventing secondary structure formation and ensuring translation at low temperatures (Yamanaka and Inouye, 1997). CspA is also expressed during sub-zero temperature growth in members of the genus *Exiguobacterium* (Rodrigues et al., 2008). *A. pavilionensis* RW1 also contains a rare branched unsaturated PLFA iC15:1Δ^4^ and other branched unsaturated PLFAs, which could regulate membrane fluidity to combat the colder temperatures found in Pavilion Lake (Los and Murata, 2004).

*Agrococcus pavilionensis* RW1 was able to metabolize a wide range of carbon compounds. These include amygdalin (**Table 3**), which has not been reported for other *Agrococcus* spp., and is surprising because amygdalin-specific glycosylases were not predicted by the genome. Amygdalin utilization is known for its distant relative *Rhodococcus kunmingensis*, an actinobacteria isolated from soil (Wang et al., 2008). Analysis of the *A. pavilionensis* RW1 genome predicts carbohydrate utilization pathways for mannose, fructose, D-gluconate, trehalose, D-ribose, and glycogen, as well as for chitin, lactate, glycerate, deoxyribose, and deoxynucleoside catabolism. Carbohydrate utilization tests for D-glucose, D-mannitol, and D-sucrose validated the metabolic potential of the *A. pavilionensis* RW1 genome (**Table 3**). *Agrococcus pavilionensis* RW1 was able to grow on many more single-carbon sources compared to other members of the genus, possibly allowing access to carbon provided by cyanobacterial mats (Breitbart et al., 2009; **Table 3**).

### Heavy Metal Metabolism and Detoxification

*Agrococcus* spp. appear to be a component of the Pavilion Lake microbialite community and potentially contribute to the detoxification of heavy metals. Such detoxification potential appears to be, an accessory feature of microbialite communities (Ruvindy et al., 2016; White et al., 2016b; Kurth et al., 2017). Heavy-metal resistance genes, particularly those for arsenic resistance and metabolism, appear to be common in freshwater microbialites in Pavilion Lake (White et al., 2016b), as well as in microbialites from Socompa Lake in the Andes (Kurth et al., 2017) and in the marine stromatolites of Australia’s Shark Bay (Ruvindy et al., 2016). We completed metagenomic-read recruitments from the 20 m Pavilion Lake microbialite metagenome (White et al., 2016b), comparing them to reads from our genome of *Agrococcus pavilionensis* strain RW1 (i.e., plasmids and chromosome). Using metagenomic-read recruitment, we confirm our *Agrococcus pavilionensis* strain RW1 is part of the Pavilion Lake microbial community (White et al., 2016b) We also obtained an assignment of previously unclassified actinobacterial sequences as 1% of metagenomic reads from the microbialite from which *A. pavilionensis* RW1 was isolated (**Supplementary Figure S2**). Metagenomic sequences recruited with the highest similarity (>95%) to the *A. pavilionensis* RW1 genome through tBLASTx (1e^-3^) were predicted to be heavy-metal resistance genes. The metagenomic-read recruitment found hits to heavy-metal resistance genes contained on both the plasmid pLC-RRW783 and in the main chromosome of the *A. pavilionensis* RW1 genome. Sequences from the Pavilion Lake 20 m metagenome match the mercuric ion reductase and arsenic resistance genes (a*rsC* and a*rsR*) in the pLC-RRW783 plasmid. That means these heavy-metal resistance genes encoded in pLC-RRW783 plasmid could be mobile and potentially could be transferred to other bacteria. Together these data suggest that *A. pavilionensis* RW1 is a source of the heavy-metal resistance genes within the Pavilion Lake metagenome (White et al., 2016b). That adds new metabolic capabilities linking a cultured isolate directly to the Pavilion Lake microbialite community.

In other microbialite studies, these heavy-metal resistance genes are predominantly recruited to *Proteobacteria* and *Firmicute* phyla. However, some sequences were found relating to *Corynebacterium*, a distant relative of *Agrococcus* (Kurth et al., 2017). It is possible that members of distant phyla are transferring these heavy-metal resistance genes around by way of the horizontal gene transfer of plasmids (e.g., pLC-RRW783). Pavilion Lake water has undetectable levels of arsenic, cadmium, cobalt, copper, and chromium, along with very low levels of zinc (0.01 to 0.03 mg L^-1^) (Lim et al., 2009). Thus, it is unclear why an organism from Pavilion Lake would carry gene clusters for heavy-metal resistance. Nevertheless, heavy metal resistance in microbialite communities appears to be an accessory metabolism feature (Ruvindy et al., 2016; White et al., 2016b; Kurth et al., 2017). The pLC-RRW783 plasmid arsenic resistance genes (a*rsC* and a*rsR*) encoded in RW1 are glutathione-dependent, which appears to be common in low-arsenic environments (Escudero et al., 2013). Mercury was actively mined near Pavilion Lake in the 1940s (Stevenson, 1940), and could have been at a higher concentration at one time, suggesting these are vestiges from that era.

Generally, heavy metals including arsenic limit microbial growth which in turn would limit the growth of microbialites. However, we find two examples of thriving modern microbialite ecosystems in the presence of high arsenic, Laguna Brava (Sancho-Tomás et al., 2018) and Socompa Lake microbialites (Kurth et al., 2017). While the remnants of heavy-metal metabolism and detoxification are present in genomes of organisms currently in low heavy-metal environments Pavilion Lake (White et al., 2016b), and marine stromatolites of Shark Bay (Ruvindy et al., 2016).

The extracellular polymeric substances within cyanobacterial microbialite mats and biofilms bind heavy metals, then concentrate and remove them from the water column (Arp et al., 1999). Cyanobacteria are the primary producers in microbialites ecosystems (Dupraz et al., 2009), and are sensitive to heavy metals (Dudkowiak et al., 2011). Any heterotroph that removes and detoxifies heavy metals as a byproduct of their metabolism would be rewarded by substrates for growth (e.g., carbon, nitrogen, phosphorus, and metals) by healthy cyanobacterial mats. The removal and detoxification of these heavy metals would benefit the entire microbial communities within microbialites because high metal concentrations would lead to eventual collapse of microbial population levels. Initially, these genes may have conferred heavy-metal resistance, but now function under other stressors. Or they are still maintained to resist heavy metal, which would limit growth at the sub-micron level in cyanobacterial mats.

However, alternative hypotheses are possible, including that initially heavy-metal resistance genes are now serving alternative functions. Heavy-metal resistance genes can have secondary roles, as in *Rhodobacter sphaeroides*, where arsenic resistance genes have higher expression under high-salt stress (Tsuzuki et al., 2011). Metagenomic sequencing of Pavilion Lake microbialites revealed accessory metabolic genes related to heavy-metal and antibiotic resistance (White et al., 2016b). The heavy metal resistance genes in strain RW1 are retained in a low heavy-metal environment because they detoxify other substrates (e.g., antibiotics). Heavy metals drive co-selection of antibiotic resistance when aquatic systems are impacted by agriculture or other anthropogenic means (Seiler and Berendonk, 2012). Resistance in heavy metals has conferred resistance to antibiotics in a complex microbiome (e.g., chicken guts) (Nisanian et al., 2014). Further experimental evidence is needed to confirm whether these genes within strain RW1 confer heavy-metal resistance or other functions. In either case, strain RW1 carries these genes on plasmids and may be involved in transferring such accessory genes (e.g., heavy-metal resistance or antibiotic resistance) to the entire microbiome of Pavilion Lake.

### Carotenoid Biosynthesis

The pathway responsible for the yellow pigmentation in the genus *Agrococcus* has not been described, although it has been suggested that diaminobutyric acid within the cell wall could impart the distinctive lemon-yellow colony color (Groth et al., 1996). However, other bacteria (e.g., *Cronobacter sakazakii*) have yellow colonies in the absence of diaminobutyric acid (Zhang et al., 2014). Actinobacterial isolates form yellow colonies and produce C_40_ carotenoids (e.g., canthaxanthin and echinenone) and C_50_ carotenoids (e.g., flavuxanthin) (Tao et al., 2007; Klassen, 2010). *Cronobacter sakazakii* strain BAA894 is a Gammaproteobacterium that produces yellow-pigmented colonies via a carotenoid biosynthetic pathway. When the *Cronobacter* carotenoid biosynthetic pathway was reconstructed and expressed in *E. coli*, the resulting colonies could produce lycopene, β-carotene, and cryptoxanthin, or zeaxanthin (Zhang et al., 2014). The production of zeaxanthin or zeaxanthin glycoside in *E. coli* changed the colony pigmentation from white to yellow (Zhang et al., 2014).

**FIGURE 7:**
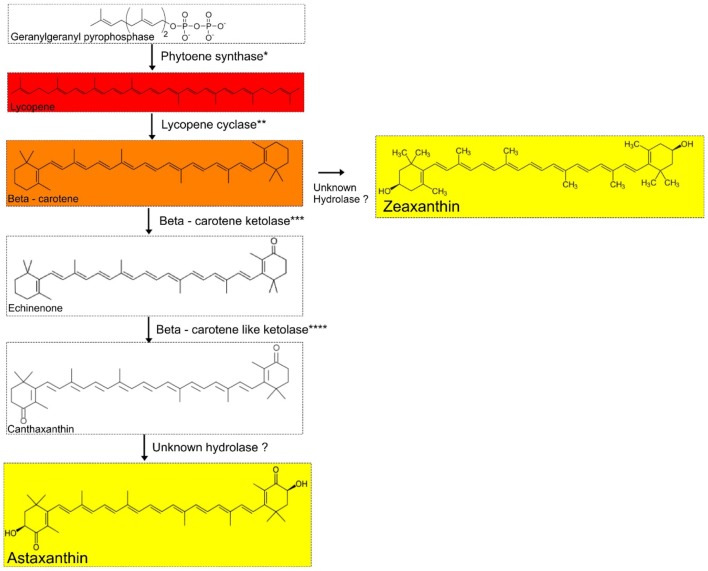
Proposed carotenoid biosynthetic pathway for *Agrococcus* spp. Isolates of *Agrococcus* spp. have the genetic potential to accumulate C_40_ carotenoids (canthaxanthin and echinenone). Hydrolases are not predicted in the genomes of *A. pavilionensis* RW1 or *A. lahaulensis* K22-21. ^∗^Phytoene synthase, ^∗∗^Lycopene cyclase, ^∗∗∗^Beta-carotene ketolase, ^∗∗∗∗^Beta-carotene-like ketolase are predicted in the genomes of *A. pavilionensis* strain RW1 and *A. lahaulensis* strain K22-21.

We screened *A. pavilionensis* RW1 and *A. lahaulensis* K22-21 for genes involved in carotenoid production; based on these results we propose a C_40_/C_50_ carotenoid biosynthetic pathway (**Figure 7**). Both *A. pavilionensis* RW1 and *A. lahaulensis* K22-21 have the genetic potential to produce lycopene, β-carotene, canthaxanthin, echinenone, and zeaxanthin, or astaxanthin (**Figure 7**). The carotenoid biosynthetic pathways include predicted genes for phytoene synthase, phytoene dehydrogenase, beta-carotene ketolase, and the second beta carotene-like ketolase that probably converts echinenone to canthaxanthin, which t implies a C_40_/C_50_ carotenoid pathway (Tao et al., 2007; **Figure 7**). Genes within the carotenogenic gene cluster of *A. pavilionensis* RW1 and *A. lahaulensis* K22-21 shared similarity to both *Brevibacterium linens*, a C_50_ carotenoid producer, and to *Cronobacter sakazakii* BAA894 (Krubasik and Sandmann, 2000).

The carotenoid biosynthetic pathway in *A. pavilionensis* RW1 and *A. lahaulensis* K22-21 is similar to that described for *Cronobacter sakazakii* BAA894 (Zhang et al., 2014). Zeaxanthin is the most likely pigment responsible for the yellow pigmentation in *Agrococcus* strains since its genome lacks the hydrolase gene necessary to convert zeaxanthin to other yellow pigments, such as astaxanthin, another yellow carotenoid that could be responsible (**Figure 7**). No hydrolase coding sequences were found in the genomes of *A. pavilionensis* RW1 or *A. lahaulensis* K22-21, which is the only type of enzyme known to convert β-carotene to zeaxanthin or canthaxanthin to astaxanthin (Klassen, 2010; Zhang et al., 2014).

The function of the yellow pigmentation in *A. pavilionensis* RW1 remains unclear, though we have excluded the possibility of phototrophy since bacteriorhodopsins and xanthorhodopsins were absent from its genome. A bacterial phytopathogen, *Pantoea stewartii*, has a phytoene synthase similar to strain RW1. It produces a yellow-pigmented carotenoid that has antioxidant properties (i.e., it is less sensitive to hydrogen peroxide stress), and it enables UV radiation protection (Mohammadi et al., 2012). The possibility that they function in photoprotection is a reasonable alternative, since the exceptionally clear waters of Pavilion Lake (due to low dissolved organic carbon) allow for high penetration of solar UV radiation (Lim et al., 2009; Lionard et al., 2012). In this way, the yellow pigment in *Agrococcus pavilionensis* RW1 may act as a kind of protective sunscreen.

Carotenoids are known signal molecules beyond their role in pigmentation or photoprotection. Carotenoids can inhibit virulence factors in pathogens such as zeaxanthin, which inhibits *Pseudomonas aeruginosa* quorum-sensing systems and biofilm formation (Gökalsın et al., 2017). *Pantoea stewartii* yellow carotenoid, while providing both antioxidant properties and UV protection, also makes its carotenoids in a quorum-sensing dependent manner via the EsaI/EsaR system (Mohammadi et al., 2012). As with *Pseudomonas aeruginosa, Pantoea stewartii* losses virulence when its carotenoids production is limited (Mohammadi et al., 2012). In the non-pathogen *Rhodococcus* sp. SD-74, carotenoids are rapidly accumulated in biofilms (∼1 week). Bacterial cell aggregation or biofilm formation may trigger their synthesis (Zheng et al., 2013). We speculate that carotenoids in strain RW1 may help to trigger biofilm formation on carbonate minerals, initializing the steps in microbialite formation. Whether the function of carotenoids in strain RW1 is beyond the colony pigmentation presented here is unknown. Further investigations are needed to put strain RW1 into carotenoid roles in photoprotection, antioxidant properties, quorum-sensing, cell aggregation, and biofilm formation.

## Conclusion

Our study provides a complete reference genome sequence for the first time from a member of the genus *Agrococcus.* Strain RW1 was isolated from a modern microbialite and possesses some features that distinguish it from previously characterized members of this genus. The presence of mobile elements, plasmids and a putative prophage in the genome implies much genetic promiscuity and could in part be responsible for high genomic plasticity as revealed by the low-gene similarities between *A. pavilionensis* RW1 and *A. lahaulensis* K22-21. The LC-RRW783 plasmid and the chromosome of *A. pavilionensis* encode genes related to heavy-metal resistance (and confer antibiotic resistance). Signatures of this encoding were also found in the metagenomic data from Pavilion Lake, confirming its presence and a potential role. In addition, the biochemical properties and physiological capabilities of *A. pavilionensis* RW1 were distinct from other members of the genus. *A. pavilionensis* RW1 possesses PLFA iC15:1Δ^4^, has β-galactosidase activity, and uses amygdalin as a sole carbon source.

Phylogenetic analysis using either 16S rRNA gene or MLST could not resolve *A. pavilionensis* RW1 and *A. lahaulensis* K22-21 as different species and placed them consistently in the same clade. However, the whole-genome analysis did resolve that *A. pavilionensis* and *A. lahaulensis* are separate species based on relatively low functional gene conservation and less than 95% amino acid identity between the genomes. The presence of many non-conserved intergenic regions in *A. lahaulensis* also supports the classification of *A*. *pavilionensis* RW1 and *A. lahaulensis* K22-21 as separate species.

One of the most surprising aspects of *A. pavilionensis* was its high growth temperature, which may reflect its descent from a population of durable generalists, as seen in the diverse habitats where the genus can be found. However, the genome of *A. pavilionensis* also shows characteristics that may reflect adaptations to its present environment (or to conditions in the recent past, as in the case of heavy-metal resistance). These include a lipid profile ostensibly suited for cold climates, the possession of cold-shock proteins, and a low-phosphorous response regulon (Pho), all of which could be of use in a cold, oligotrophic environment. Other features may represent pre-adaptations; such as the carotenogenic gene cluster whose products could provide photoprotection in the clear water column of Pavilion Lake.

Strain RW1 was investigated for its potential role in Pavilion Lake microbialites, including candidate processes (e.g., ammonification and filament-like growth) by which actinobacteria may contribute to microbialite formation. These results provide a blueprint for future efforts to characterize stress response, pigment synthesis, and phage interactions in this widespread genus. Transcriptomics (including single cell) (Gavelis et al., 2015) and proteomics (White et al., 2016a; Callister et al., 2018) should be used in future experiments with strain RW1 to further elucidate functional profiles within the genome. *A. pavilionensis* strain RW1 represents a model system for further study of non-photosynthetic pigmented heterotrophic bacteria present within modern microbialites and microbial mats. Further exploration of microbialite-associated taxa is crucial to the understanding of these ecosystems and should include not only those driving the formation of the microbialite but also those contributing to the overall development and health of the community.

## Author Contributions

RW designed the study, collected and plated the isolate, performed growth studies, extracted DNA, prepared libraries, assembled and annotated the genome, and performed comparative genomic and phylogenetic analysis. SS performed PLFA, with financial support from GS. EG performed culturing experiments. RW preserved cells for scanning electron microscopy, which was imaged by GG. RW and GG wrote the manuscript. All authors participated in the manuscript drafting process.

## Conflict of Interest Statement

The authors declare that the research was conducted in the absence of any commercial or financial relationships that could be construed as a potential conflict of interest. The reviewer AL and handling Editor declared their shared affiliation at time of review.

## Supplementary Material

FIGURE S1 Putative prophage genome from RAST annotation. The gray represents genes of hypothetical function. The red gene (label 1) is hypothetical phage gene homologous across strain RW1 and the representative phage/bacterial comparison genomes. The light blue (labeled 16) is phage terminase and tail proteins. The numbers represent genome locations.

FIGURE S2 FR-Hit recruitment plot of Pavilion Lake 20 m microbialite reads (∼7.5 Million) to *A. pavilionensis* strain RW1 isolated from 20 m microbialites and to *A. lahaulensis* strain K22-21.

TABLE S1 Final assembly statistics. ^∗^Published in White and Suttle (2013).
