# Supplementary figures and images for: The Complete Genome and Physiological Analysis of the Microbialite-Dwelling Agrococcus pavilionensis sp. nov; Reveals Genetic Promiscuity and Predicted Adaptations to Environmental Stress
Source: Front Microbiol. 2018 Oct 15;9:2180. doi: 10.3389/fmicb.2018.02180 (PMC6196244; doi:10.3389/fmicb.2018.02180)

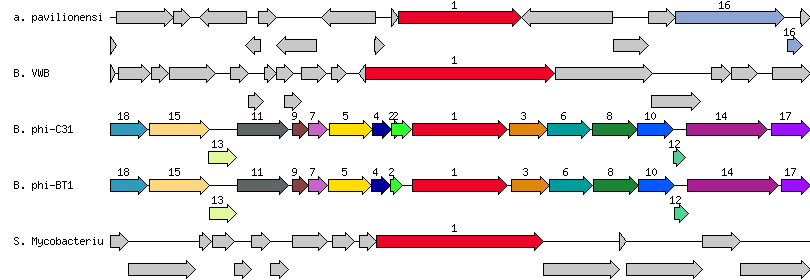

Supplement: FIGURE S1 — Putative prophage genome from RAST annotation. The gray represents genes of hypothetical function. The red gene (label 1) is hypothetical phage gene homologous across strain RW1 and the representative phage/bacterial comparison genomes. The light blue (labeled 16) is phage terminase and tail proteins. The numbers represent genome locations. [file Image_1.PNG]

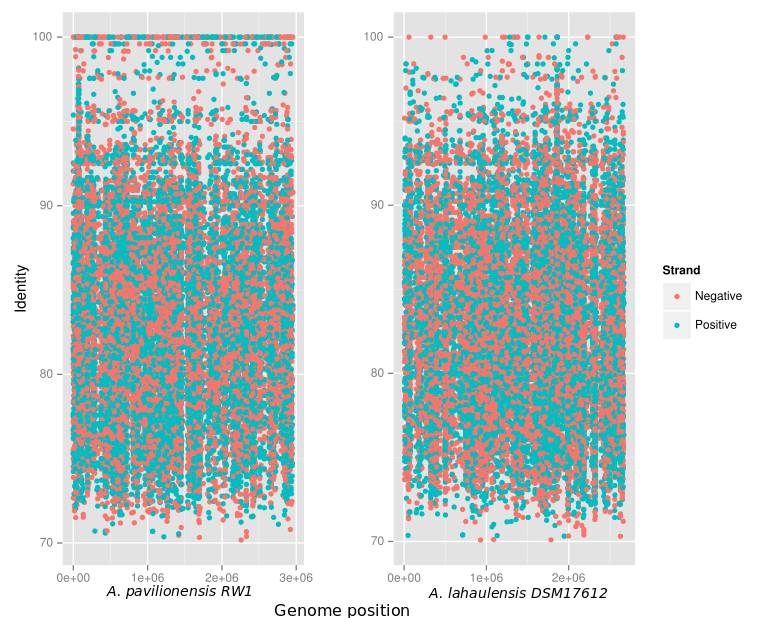

Supplement: FIGURE S2 — FR-Hit recruitment plot of Pavilion Lake 20 m microbialite reads (∼7.5 Million) to A. pavilionensis strain RW1 isolated from 20 m microbialites and to A. lahaulensis strain K22-21. [file Image_2.PNG]
